# Causal explanations of miscarriage amongst Qataris

**DOI:** 10.1186/s12884-017-1422-5

**Published:** 2017-07-27

**Authors:** Susie Kilshaw, Nadia Omar, Stella Major, Mona Mohsen, Faten El Taher, Halima Al Tamimi, Kristina Sole, Daniel Miller

**Affiliations:** 1 University College London-Department of Anthropology, 14 Taviton Street, UCL, London, WC1E6BT UK; 2 Weill Cornell Medicine-Qatar, Doha, Qatar; 3 Women’s Hospital-Hamad Medical Corporation, Doha, Qatar; 4 University of Oslo, Oslo, Norway

**Keywords:** Miscarriage, Qatar, Causation, Risk, Preventive measures, Qualitative research

## Abstract

**Background:**

Despite its commonality, there is a paucity of literature on miscarriage in non-Western societies. In particular, there is little understanding of how people ascribe cause to miscarriage. This research sought to gain an in-depth understanding of notions of miscarriage causality and risk amongst Qataris.

**Methods:**

The study adopted an exploratory descriptive qualitative approach and collected data during 18 months of ethnographic research in Qatar, including semi-structured interviews. The sample includes 60 primary participants (20 pregnant women and 40 women who had recently miscarried), and 55 secondary participants including family members, health care providers, religious scholars and traditional healers. Informed consent was obtained from all participants. Primary participants were interviewed in Arabic. The interviews were audio recorded, transcribed and translated into English. Data was analysed using an inductive thematic approach, which involved identification and application of multiple codes to different text segments. Data were encoded manually and examined for recurrences across the data set. Similar quotations were grouped into subcategories and further categorized into main themes.

**Results:**

A number of key themes emerged, revealing Qatari women attributed miscarriages to a number of factors including: supernatural forces, such as God’s will and evil eye; lifestyle, such as physical activities and consuming particular substances; medical conditions, such as diabetes; and emotional state, such as stress, and emotional upset. Resting, avoiding stress and upset, maintaining healthy diet, and spiritual healing (*ruqyah*) are seen as a means to avoid miscarriage.

**Conclusion:**

Practices and beliefs around miscarriage are embedded in social, cultural, religious and medical frameworks. Understanding the socio-cultural context and understandings of explanatory theories can enhance health care providers’ understandings, resulting in improved communication and care.

## Background

Medical anthropology has shown that ideas about health are cultural and are embedded in a matrix of wider cultural systems of value. Understandings of illness causation derive from the underlying intellectual orientation of a cultural group. Anthropologists have shown that the experience of suffering is not only personal: it is deeply influenced by culture and its particular moral world [1]. Pregnancy and birth are culturally patterned, and women’s knowledge, beliefs and behaviours are shaped by cultural context [2]. Anthropologists have exposed the ways in which reproduction is embedded within larger social, cultural, economic, and political relations and forces (for summary see [3]; for examples see [4–6]). This paper is derived from a larger research project whose objective was to collect nuanced and detailed data about pregnancy loss as it is contextualized in women’s lives. This paper focuses on Qatari causal explanations of miscarriage: it is our hope that this will stimulate discussions more widely about the importance of socio-cultural context on women’s experience of miscarriage. Miscarriage is the early spontaneous loss of pregnancy prior to the point of expected foetal viability [7] usually before 20 weeks of gestation, although exact threshold varies depending on context [8]. Approximately 20% of clinically recognized pregnancies worldwide end in miscarriage [9]. However, the actual rate of miscarriage is even higher since many women have early miscarriages without realizing that they are pregnant [10]. Thus, miscarriage is a common women’s health experience. In EuroAmerican settings miscarriage is often taboo and can be seen as a source of guilt and shame [11] and produce a variety of psychological distress outcomes, including grief, anxiety, depression and guilt [12]. Despite its prevalence, the experience of miscarriage has been relatively understudied. The existing literature on miscarriage tends to focus mainly on the experiences of Western women [13–15], with few examining the issue in other cultural contexts [16–18]. Existing research suggests that cultural context dramatically impacts the experience and management of miscarriage.

Qatar is an Arab country in the Middle East with Islam being the predominant religion. The population is 2.2 million, with Qatari citizens representing a 10% minority with the rest comprised of migrant workers from all over the world [19]. Concerned about demographic imbalances and eager to increase capacity of the local population, the state has focused on numerous pronatalist measures. The Qatari Population Policy 2009 promotes higher fertility rates among Qataris [20] through encouraging and facilitating marriages among Qatari citizens, providing financial incentives to support housing and having a big family, and recruiting religious figures who emphasise the religious duty of marriage and childbearing [21]. Women are encouraged to have many children and the total fertility rate of Qatari women is one of the highest in the Arab Gulf States. Yet, at the same time the rapid socio-economic development in Qatar has provided women with greater opportunities for education and employment [22].

Qatari women experience multiple pregnancy events [23] and thus, are at risk of miscarriage and multiple losses. Furthermore, despite the lack of concrete statistics, it is likely that rates of miscarriage are increased in Qatar due to the popularity of consanguineous^1^ marriages, which have been found to have a significant association with negative pregnancy outcomes in other contexts [24]. Recent research has revealed that the rate of consanguinity in the present generation is 51% [25].

Academic research on health in the Middle East is particularly lacking [26]; healthcare professionals agree that there is a lack of information about Qatari health generally and women’s health in particular [27]. There has been no anthropological research on pregnancy loss in the region. The overall research question was to understand the impact of cultural context on how miscarriage is experienced and understood by comparing two different cultural contexts: UK and Qatar. This paper focuses on an aspect of the larger matrix by investigating which factors Qataris understand to cause miscarriage and how are these linked to the wider socio-cultural context. This study responds to a gap in academic and medical knowledge by investigating local notions of miscarriage causation. In most individual cases, the cause of a miscarriage remains unknown [28] often leading to a sense of uncertainty, which can be linked to a search for meaning in making sense of the loss. Little is known about lay perceptions of miscarriage causation [29], due, in part, to the sensitive nature of miscarriage [13, 30]. Understanding how people understand the determinants of health and illness is important for health professionals and for public health and health promotion efforts. Furthermore, a disconnect between professionals’ beliefs and illness models and those of their patients, may result in the latter unlikely to engage with strategies to manage health. This dynamic has been a focus of anthropological research with extensive literature focusing on lay concepts and causal theories of illness. This body of work provides evidence of the complexity and interconnectedness of ideas about health and illness. Our findings reveal certain specific aspects of Qatari miscarriage causation, which have similarities to other cultures of Eastern Arabia. Thus, the paper aims to provide detail about Qatari notions of miscarriage causation to provide better understanding of lay models, which can inform improvement in care, management and support for miscarrying women can be provided.

## Methods

The data for this paper is based upon 18 months of ethnographic research in Qatar (May 2013–January 2014), which was part of a larger comparative investigation into miscarriage in Qatar and the UK. The overall research explores how cultural context impacts the experience of pregnancy loss by comparing two cultural groups and considering the social and cultural constructions of the body and their influence on illness representations [31]. The ethnographic research combines a study of women who are experiencing pregnancy and pregnancy loss, with additional key interviews with those involved in lives of these women (clinicians, religious experts, traditional healers, family members) and participant observation. Listening to what women themselves have to say about their health and wellbeing is of vital importance to policy making [3] and, hence, was the focus of the research.

### Study design

The project utilized in-depth, embedded and analytic ethnography [32, 33] with the gradual accumulation of data through observation and the inductive analysis of these data [34]. The approach involved interviews, conversation, direct observation and participant observation: a commitment to a depth of encounter is uniquely suited to this subject because of its intimate and personal nature. During the course of the fieldwork, observation of selected clinical and non-clinical sites provided additional information and contextualised the interview data. In the hospital these included observing sonogram sessions and attending clinics and meetings. Participant observation included: attending clinical and sonogram sessions with pregnant participants, visiting participants in their homes, participating in Qatari mothers’ groups, and accompanying women to traditional healers. Observations and informal interviews occurred mainly in opportunistic ways over the 18 months period. Our approach makes use of a framework for multisited ethnography [35] and thus, as the research progressed, we were informed by our participants and followed miscarriage across sites including: the home, the mortuary, the mosque, the graveyard, traditional healer’s clinics, and religious sites.

### Participants

The sample includes: 60 primary participants (20 pregnant women and 40 women who had recently miscarried). Although primarily interested in miscarriage, unlike in other parts of the world (i.e. see [36, 37]), little is known about pregnancy in Qatar. The authors felt it important to develop a foundation of knowledge around the cultural shaping of Qatari pregnancies to better understanding of *what* is lost when a pregnancy is unsuccessful, thus a sample of pregnant women was included. In particular, ideas of what constitutes a “good” or “normal” pregnancy or pregnant body [38] as well as theories of conception were explored. Women with more than three consecutive pregnancy losses at any gestation were excluded from the study.^2^ Fifty-five secondary participants provided additional material on Qatari pregnancy, birth and loss. Interview and interactions with husbands (n7 from miscarriage cohort) and family members (n16 from miscarriage cohort, n13 pregnancy cohort), 13 health professionals, 2 religious scholars, and Qatari 4 traditional healers were included.

### Recruitment

Participants were recruited from the hospital outpatient department, inpatient rooms, and the early pregnancy unit. The Qatar based Arabic speaking research associates approached potential participants (Qatari women 18–50, miscarried in the past 6 months prior to 20 weeks gestation or less than 20 weeks pregnant) once identified and referred by collaborating healthcare providers and having expressed interest in participating. The research associates explained the study and participants were given printed information sheets. Project flyers and posters in the hospital outpatient clinics also provided a recruitment route with potential participants contacting the research associates directly. Details of the study and involvement were discussed and the participant given time to review the information sheet and ask questions. If the woman expressed a desire to proceed, verbal informed consent was obtained and the participant was entered into the study. Given the sensitive subject and the rich detailed descriptions of the interviewee and their life to be collected, in accordance with Qatari Law recommendations regarding protecting the sanctity of the individual’s privacy [39], signed consent was waived [in accordance with the code 45 CRF 46.117(c)(1)] [40]. Health professionals from the clinical site and religious leaders were approached and asked if they would volunteer to participate. Traditional healers were identified by participants and were approached directly. Husbands and other family members were recruited through the participant for reasons of cultural sensitivity and notions of privacy.

### Data collection

Interviews were conducted in the hospital or at a participant’s preferred location (e.g. their home or a café). Interviews were conducted in Arabic and were 1–2 h in duration using a semi-structured interview script, which acted as prompts to allow for significant issues to emerge [41]. Different interview scripts were used for the two cohorts. Both interview scripts collected background information, explored motherhood and women’s role in Qatari society, and investigated topics related to pregnancy, notions of risk (including ideas around miscarriage and foetal harm). The miscarriage interview script also covered details about the miscarriage, specific causation explanations, and feelings about the loss. Ideas of risk, pregnancy and miscarriage were covered in both, with most women speaking about their reproductive experiences generally. The data presented here are derived from the questions around risk, cause of miscarriage and preventative measures including: Why does a woman miscarry? Why do you think you miscarried? Is there anything a woman can do to prevent a miscarriage? Further information was derived from more informal discussions about risk and cause.

Interviews were recorded by digital voice recorder and transcribed and translated into English soon afterwards, whilst retaining key words and phrases in Arabic. For accuracy, the two bilingual research specialists crosschecked several transcripts. The lead investigator read the interviews immediately and discussed any points for clarification and follow-up questions were developed. When possible, the lead investigator conducted interviews in English or in Arabic (with the assistance of the research specialists). Wherever possible contact with participants was maintained as is typical of in-depth ethnographic research to allow for follow-up and more informal discussions and observations. This approach allows for gradual accumulation of data and slow inductive analysis with further interviews and fieldwork interactions shaped by emerging understandings. Such an approach means developing rapport and gaining deeper insight into how miscarriage is understood in the wider social and cultural matrix. Amongst the pregnancy cohort twelve participants were interviewed one to two times, five were interviewed between six and nine occasions. In the miscarriage cohort seven participants were interviewed on three to six occasions and others were interviewed once to twice. The number of times a participant was interviewed was based on their interest and availability. To preserve anonymity, identifying participant details have been altered or omitted. Additional data included documents such as patient flyers and posters, detailed fieldnotes and fieldwork diaries.

### Data analysis

Analysis of the qualitative data sets generated by the research was through a process of thematic coding and analysis. The basis of analysis was deep familiarity with the data and an ongoing, recursive process of reading, discussing and reflecting on the data as it is collected [42, 43]. Through ongoing discussions and regular team meetings we reflected on developing understandings, and collected additional data as necessary. Emerging understandings and the identification of key knowledge gaps informed further interviews and fieldwork interactions. We identified themes and put them into categories, with higher order categories emerging in line with the constant comparative method [44]. The value of this process of data collection and analysis is that it yields in-depth, nuanced data from multiple perspectives, which captures the complexity of participant’s experiences and the networks of meaning within which they are embedded.

Quantitative data such as age, employment status, number of pregnancies, number of miscarriages, and number of children for all participants was extracted and entered into tables. Data was organised into categories, interrelated with the abstracted themes and then analyzed for identification of group patterns. Simple descriptive statistics were used to examine the patterns of socio-demographic data.

## Results

- Quantitative: The socio-demographic distribution across age, education level, and employment status for the miscarriage and pregnancy participants are illustrated in Table 1. Primary participants were Muslim Qataris with an average age of 26 years for pregnant participants and 33 years for those with a miscarriage. They were generally educated; more than 50% of pregnancy and miscarriage cohorts achieved at least undergraduate education. The majority (60%) of both groups are employed.
- Qualitative: A number of key themes emerged around narratives of miscarriage causation and risk: supernatural factors, lifestyle and diet, medical conditions, and emotional state of the mother.

**Table 1:** Background characteristics of primary study participants

Characteristic	Miscarriage (*n* = 40)	Pregnancy (*n* = 20)
Maternal Age (yrs)		
20–29	15 (37.5%)	14 (70%)
30–39	21 (52.5%)	5 (25%)
40–49	4 (10%)	1 (5%)
Educational Level		
Illiterate	3 (7.5%)	0 (0%)
Up to high school	12 (30%)	9 (45%)
Higher education	25 (62.5)	11 (55%)
Employment		
Employed	24 (60%)	14 (70%)
Unemployed	16 (40%)	6 (30%)
Number of Live Births		
None	7 (17.5)	6 (30%)
1	6 (15%)	4 (20%)
2–4	12 (30)	6 (30%)
≥ 5	15 (37.5%)	4 (20)
Total Number of Pregnancies		
1–2	11 (27.5%)	10 (50%)
3–4	5 (12.5%)	4 (20%)
≥ 5	24 (60%)	6 (30%)
Total Number of Miscarriages (none-consecutive)		
Non	0 (0%)	12 (60%)
1–3	34 (85%)	5 (25%)
4–6	6 (15%)	3 (15%)

### Explanations of cause of miscarriage

Miscarriage was generally not silenced in Qatar and was often described by participants as “normal”: participants explained that they were familiar with other women who had miscarried. Given the high fertility rate, Qatari women experience a number of pregnancies and many experience adverse outcomes: miscarriage was seen as not an unusual event in a woman’s reproductive life. Huda,^3^ a 35-year old woman with three children, explained that she was not upset or worried after her first miscarriage:

The first miscarriage was normal for me because my mom miscarried and many women who I know also had miscarriages and then they had children… My mother in law told me that it always happens with women because of tiredness and once you had children before you will have again so it is not a big deal.

Reem, a 35-year old woman with five children who had recently miscarried for the first time, concurred:

For us it [miscarriage] is normal, but for someone who miscarries a lot they feel sorry for her.

Whilst miscarriage was not seen as particularly unusual, participants speculated about cause of miscarriage. They reported a variety of possible causes, which can be categorized into the following headings: supernatural, lifestyle, medical conditions and chromosomal abnormalities, and psychological.

### Supernatural

Participants commonly attributed their loss to supernatural forces including God’s will and the activities of supernatural beings.

#### God’s will

The ultimate reason for miscarriage is God’s will, according to participants. Khoulood, a 42-year-old participant with one child who had recently miscarried, explained:

*Allah’s decision is always good. Allah knows his people and what is good for them and what is not. Allah saw that this baby is not good for me so he took it back… He may be meant to be abnormal or disabled or may grow up to be a tyrant and will kill me or kill his father or he may grow up to be a corrupted person. There must be wisdom of his death. I believe in Allah and I accept whatever comes from him.*

Like Khoulood, others explained that if the baby had lived he/she would have been born with disabilities or cause problems within the family. By referring to ‘*Qadr’,* a decree or preordainment of God; participants understood miscarriage as an ordeal chosen by God. Participants suggested this allows one to accept miscarriage and to see it as beyond their responsibility. Whilst participants explained that God’s will is the ultimate cause of miscarriage, they attributed other factors as immediate or natural causes.

#### Evil eye

Evil eye is prevalent in Qatari explanations of miscarriage causation with 33% of miscarriage participants referring to it and is commonly activated in the absence of certainty of cause. Evil eye is cast, often by women, “who may be jealous, envious or simply wicked, and who intentionally or unintentionally harm” by a glance [45]. Awatif, a 36-year-old miscarriage participant with four children, reported her miscarriage occurred immediately after her parents and in-laws came to know about her pregnancy:

*The problem is if people know that I am pregnant, something wrong will happen to me, if anyone talked about my pregnancy I am sure something bad will happen to me.*

Once a pregnancy becomes known it is vulnerable and at risk of evil eye. Some participants indicated that the older generation and those who were uneducated place more emphasis on evil eye, as illustrated by Sameera, a 44-year-old miscarriage participant who has six children and two miscarriages:

*Yes, they say it is evil eye … but in general, people are educated now and they know that there must be a reason that has caused the miscarriage. They suggest ‘Ruqyah’ to treat evil eye. Old people believe in evil eye; they worry more. When I told my mother that I am pregnant she told me, “Don’t tell anybody.”*

As suggested by Sameera’s mother, a common method for protection against evil eye is non-disclosure or concealment of the pregnancy. Participants referred to ‘*ruqyah,’* the recitation of certain verses of Quran and good supplications, as a means to protect oneself from and curing illness caused by evil eye. Some perform ‘*ruqyah’* themselves, whilst the majority seek treatment from a religious person known as ‘*motawa’ or ‘sheikh’.*

Despite widespread belief in evil eye, a few participants believed that others over-emphasised its role. A religious scholar confirmed the prevalence of evil eye in illness causation, including miscarriage, yet warned against its overuse:

*The eye is real and it has been narrated in ‘hadiths’ that the eye may lead a man to the grave or the camel to the pan [it may cause the death of someone and make the impossible things possible], but people now exaggerate the eye. They refer any problem that happens to them to the eye, though it may be due to other reasons. For example, maybe it is our unhealthy food that is causing this problem… But definitely, a woman with many births could be cursed especially if another woman who is infertile looked at her belly, so she may eventually miscarry. Absolutely, the evil eye is like an arrow that comes out of the eye of the jealous person.*

The *Sheikh* outlined both the prevalence of and over-emphasis on evil eye in illness causation. Noting that fertile women attract evil eye, the *Sheikh* re-iterated what was commonly expressed in interviews: that envious, but often unaware, infertile women are the most common source of evil eye. The husband of Khadeeja, a 28-year-old miscarriage participant concurred: *“the evil eye is from an infertile woman”.*

#### Possession by jinn

Three participants spoke about possession by jinn and considered it a cause of infertility and pregnancy complications. *Jinn* are supernatural creatures in Arabic and Islamic mythology, with possession occurring when a *Jinn* enters a human to cause mischief [46]. Haleema, a 33-year-old participant who had recently suffered her second miscarriage, believed herself to be possessed by a *jinni* that visits her in her dreams. In love with her, the *jinni* caused the death of her baby to alienate her from her husband. Khoulood, a 42-year-old with one child had experienced a miscarriage when she stopped reciting the Quran and rubbing her belly with blessed oil, according to her sister. Ceasing these practices had allowed the *jinn* to enter her womb and remain there:

*If the jinn is settled in the uterus it will prevent pregnancy; many people are possessed and this possession caused them to miscarry so they seek help from religious leaders and from Quran.*

Pregnant women are particularly vulnerable to *Jinn* and thus, participants suggested that one should protect oneself by reciting prayers and remembering Allah.

### Lifestyle: Activity and diet

Participants reported that carrying out strenuous work, fatigue, and travel are potential causes of miscarriage; exhaustion was a particular concern. Participants indicated that women in professional employment, requiring long working hours, exertion, movement and/ or travelling are more at risk of miscarriage. Participants commonly suggested that a pregnant woman should avoid strenuous activities, should rest and sleep. Wadha, a 34-year-old teacher with five children attributed her miscarriage to exhaustion and suggested rest as a protective measure:

*It is because of tiredness ….I think having rest is important, may be if I rested I wouldn’t have miscarried.*

Najah, a 33-year-old participant who had miscarried her first pregnancy, explained:

*I blamed myself, maybe my work rhythm is fast and I didn’t slow down… I work in communications; we have too much work… we move a lot…we have a lot of movement and pressure.*

Huda, a 35-year-old miscarriage participant with three children told us of her long work hours:

*Maybe life has changed and the woman has more responsibilities now. She goes to her work and then comes back and sometimes she is tired. Many things have changed: the life style has changed.*

The above quotation reflects changes in the expectations and responsibilities placed on contemporary Qatar women including recent government initiatives focusing on empowering women and involving them in the state’s development plans [22], particularly though an increased role social and economic life; expectations that often conflict with family responsibilities and reproduction. Work and associated physical activity were seen to cause stress and tiredness, which negatively impacted pregnancy.

Lifting heavy objects is a risk factor cited by 25% of the pregnant participants and was the second most common cause in this category amongst miscarriage participants (25%). However, none of the miscarriage participants reported carrying a heavy object during her pregnancy, suggesting it is identified as a general risk rather than a specific cause of the particular miscarriage. Noora, a 42-year-old miscarriage participant with six children, suggested this as a central feature of explanations of cause:

*In Gulf region countries we always say that a woman miscarries because she lifted a heavy object: this is the most common theory. She carried something heavy or she overworked or slept with her husband. We don’t consider the medical or biological reasons... They asked me when I had the miscarriage; “did you carry something? What did you do?” Even though the woman doesn’t do any work at home.*

Lifting something heavy is emphasised by mothers and grandmothers, a generational difference possibly due to the fact that household tasks are now performed primarily by maids and other domestic workers. However, some suggested Qatari women were still at risk due to household labour in some contexts. The mother of a miscarriage participant discussed “domestic abuse”:

*Domestic abuse is also one of the reasons; they give her so much work more than what she can afford. I know a woman who got married young and her mother-in-law was abusing her by letting her do a lot of house work, such as carrying buckets of water from one floor to another while she was pregnant.*

Referred to by three miscarriage participants, “domestic abuse” referred primarily to extended family members burdening a woman with excessive and strenuous household tasks, linking it with heavy lifting and exertion. Thus, notions of blame are shifted to the woman’s marital family.

One of the key areas of risk was in relation to diet, including: consumption of caffeine, fatty foods, “junk” foods, and certain plants and herbs [38]. Participants reported a number of herbs and plants that caused miscarriage including: red seed, cinnamon, ginger, thyme, fenugreek, black seed, sage, papaya and pineapple. These herbs and plants are considered uterine stimulants and are commonly used in the final stage of pregnancy to induce labour, or following birth or miscarriage to cleanse the uterus and expel blood and tissue. A relevant related research finding was the commonality of the use of herbs, plants and traditional medicine amongst Qatari women [38].

### Medical conditions and chromosomal abnormalities

Sixty-three percent of miscarriage participants cited medical conditions in relation to explanations of cause: these are categorised into problems with the foetus, including genetic or chromosomal abnormalities, and maternal causes such as: immunological causes (Rh incompatibility), maternal illness, uterine abnormalities (e.g. fibroids and overstretched uterine cavity), a “clot in the placenta”, exposure to radiation and/ or medications, and advanced age. Women reported that genetic or chromosomal abnormalities of the foetus might cause miscarriage, often referring to information that they had received from a clinician, or read in hospital flyers, or gleaned from the Internet. Genetic causes were emphasized by one of the clinician participants:

*International studies prove that 60% of miscarriage are due to abnormalities and, for me, I believe that this is from God because if all the 60% didn’t abort, imagine how much the number of abnormal human beings would be living on the Earth. Once I tell the patient this, she keeps quiet and she thanks God.*

The clinician emphasises foetal abnormality, but entwines this with local knowledge of God’s will as the ultimate cause of miscarriage.

Four miscarriage participants cited a “blood clot in the placenta.” Amna, a 30-year-old miscarriage participant with two children, attributed her own loss to radiation exposure, yet suggested other causes of miscarriage:

*(Why does a woman miscarry?) For many reasons: like congenital deformities or a clot in the placenta. I think a clot in the placenta is the cause of most pregnancy losses… I heard that if a pregnant woman doesn’t take Aspirin she will lose the baby.*

A blood clot in the placenta can stop the blood flow to the baby and thereby deprive nourishment, leading to foetal death. Women reported that health providers recommend taking Aspirin as a preventative measure.

Non-communicable diseases or infections were mentioned as medical conditions that cause miscarriage. Some cited “diseases” or “viruses” in general; others were more specific and referred to particular conditions such as urinary tract infections, high blood pressure, toxoplasmosis, and diabetes. The latter condition was widely cited, reflecting its high prevalence in Qatar (16.7% in adult Qatari population) [47]. A number of participants reported previous diabetes-related pregnancy complications and/or losses. Jameela, a 33-year-old diabetic woman who had experienced two consecutive miscarriages said:

*Diabetes has a big role. My previous miscarriage was because of diabetes… and my pregnancy ended.*

Other causes advanced by miscarriage participants included adverse effects of medications and radiation as the result of x-rays. Women indicated that pregnant women should not be exposed to radiation or take medications, particularly in the first trimester. Three primary participants identified the cause of their miscarriages as having Rh incompatible blood types with their husbands. Uterine abnormalities were discussed as a potential cause for miscarriage, such as endometriosis or a misshapen uterus. Awatif, a 36-year-old miscarriage participant who had experienced four losses and had five children, considered having multiple pregnancies as a risk factor:

*Because when the lady gets pregnant many times, this will weaken the uterus. I think this is the most [common] cause* of miscarriage

Multiple pregnancies and weakening of the uterus can be linked with another risk factor: advanced age. Thirty-three percent of miscarriage participants reported that advanced age plays a central role in causing miscarriage. Noora, a miscarriage participant who had six successful cesarean deliveries, but had recently miscarried her seventh pregnancy at 42 years old commented:

*Age plays a role. It was risky for me to get pregnant at this age. I asked the doctors before I decided to get pregnant and they all said: “it is not recommended.”*

Undeterred, Noora consulted three clinicians following her miscarriage, hoping one might re-assure her or suggest management strategies if she were to become pregnant*.* During a visit to Noora’s house we met her husband who endorsed the clinical view that she should not expose herself to such risk, adding that to pursue having more children he could marry again. Noora did not want her husband to take an additional wife and so was eager to become pregnant. A desire to have many children despite potential risk emerged from interviews with pregnant Rahaf, a 39-year-old diabetic mother of six children, who had suffered two miscarriages and a stillbirth. Advised by her clinician to undergo tubal ligation to avoid future pregnancies, Rahaf refused. There was ambivalence around risks associated with advanced maternal age. Some participants indicated that age has no effect and provided anecdotal evidence in support:

*My mother in law was two and a half months pregnant at my wedding… she was over 45-years-old, maybe 48… Bedouins will still have children even if they are old.*

In addition to the above-mentioned causes, accidents, “slipping,” excessive sex, and conceiving by means of Assisted Reproductive Technologies (ARTs) were also mentioned as potential causes of miscarriage. When Sameera’s mother warned her not to disclose her pregnancy (see above), she responded:

*I told her, “but mother I have six kids and I got pregnant normally not by treatment.” She said, “even though, don’t inform anyone”.*

Pregnancies resulting from ARTs are seen as making a pregnancy particularly vulnerable to the other causes mentioned above.

### Emotional state, stress and distress

Miscarriage is commonly attributed to psychological upset of the mother. A woman who suffers significant work-related or personal stress, or distress caused by bad news or a shocking event is at risk; as illustrated by Moza, a 28-year-old woman with no children after miscarrying her two in vitro fertilization (IVF) pregnancies said of her sister’s miscarriage:

*Yes, my sister miscarried once and it was due to stress. A woman started talking rubbish behind her back, so she got upset and angry and the same day she miscarried.*

Being agitated, angry or distressed is thought to be potentially damaging to a pregnancy, according to participants. Distress following the death of a loved one was commonly cited as causing pregnancy complications. Sara, a 30-year-old woman with two children, attributed her miscarriage as well as that of her sister to deaths in the family:

*My sister and I went through a similar situation: our uncle passed away recently and I was very sad for his loss… My aunt had a heart attack and died immediately. She visited us a day before her death, so when my sister heard the news she was shocked…. Immediately after my sister heard about my aunt’s death, the baby died. I don’t know if this has scientific evidence but these were two situations that we both experienced.*

Sara expresses uncertainty about the “scientific” nature of this connection, but sees a causal relationship between the stress and shock and the miscarriages. Similarly, the husband of one of our miscarriage participants said:

*She probably had the miscarriage because she was under emotional stress. Her eldest sister passed away when we were in London during our summer vacation.*

Other difficult situations can also affect pregnancy. Amna, a 30-year-old with two children who had recently miscarried said that stress, “may be a probability” of miscarriage:

*My mother in law was pregnant when she got divorced so she miscarried. So I don’t know but I think the psychological condition of the mother affects the pregnancy.*

As indicated above, a pregnant woman’s emotional state is thought to impact the safety of her pregnancy. Pregnant women are seen as potentially vulnerable: Stress, shock, and upset are risk factors that may negatively affect the pregnancy and cause miscarriage.

## Discussion

The research emphasised that causal explanations of miscarriage is influenced by cultural context. Lock [48] calls on us to “contextualize and embed bodies in time and space, thus destabilizing that which is assumed to be essentially universal, ‘natural,’ and readily standardisable, and bringing to the fore inextricable entanglements among history, the social/political, and the material.” The findings emphasise the importance of “local moral worlds” [1] and how they give rise to local understandings of miscarriage. Qatari explanation had some differences and some similarities to what we know of perceptions of cause in other contexts. Research in Western contexts has suggested that miscarriage can impact a woman’s sense of self-worth and lead to grief and anxiety due to the loss of a potential child but also motherhood and ideal feminine image [49]. A large US survey [11] found that respondents (55%) erroneously believed that miscarriage is a rare complication of pregnancy, with the majority believing that it occurred in 5% or less of all pregnancies. In contrast, pregnancy loss in Qatar is normalized, not necessarily traumatic and does not commonly elicit a grief response. Miscarriage is understood as caused by God: it happened for a reason and may be part of a process of trial through hardship. This points to the centrality of faith: understanding miscarriage in Qatar must take into account the role of Islam. According to Qatari causal explanations, God’s will is the ultimate cause with additional possible causes: a common philosophy of attributing cause to illness and misfortune throughout the world, as outlined in Evans Pritchard’s influential work [50].

Research has found widespread misconceptions about causes of miscarriage [11] with common causes including: a stressful event (76%), lifting a heavy object (64%), previous use of an intrauterine device (28%), or oral contraceptives (22%), not wanting the pregnancy (23%). Whilst there was some overlap with Qatari understandings, with both cohorts seeing a stressful event or lifting a heavy object as a common causes, Qatari explanations of causality also differed considerably. Evil eye and possession by *jinn* is perceived to be a common cause: limiting women publicizing pregnancies. Belief in evil eye is prevalent throughout the Middle East [51–53] and is a particular concern around issues of fertility and reproduction, reflecting the centrality of reproduction in Qatari society. Thus, pregnant women are vulnerable by their very nature: their pregnancy illustrates their fertility, which may attract evil eye. Clinical teams should understand Qatari women’s desire to conceal their pregnancies, particularly in relation to any necessary communications with employers, family members and in ensuring mechanisms for healthy pregnancy practices.

In Qatar, pregnancy is considered a vulnerable state [38]. Participants outlined certain risky behaviours and activities that can potentially cause harm for pregnant women and suggested avoiding them. However, in light of the escalating emphasis on and rates of female employment and higher education in Qatar, avoiding the risks associated with work, such as tiredness, workplace stress and physical activity can be difficult. Narratives of miscarriage risk and the work environment reveal tensions around contradictory models promoted for women. The workplace is often viewed as potentially risky for pregnant women, something which clinical teams should be aware in order to help pregnant women effectively manage their responsibilities. The dietary perceptions and food taboos and their impact on pregnancy is embedded in wider cultural ideas about food, herbs, and traditional medicines. Pregnancy-related food habits are known in virtually all societies as written and unwritten social rules [54, 55]. The majority of these risk factors were transferred as part of oral history mostly from mother to daughter or grandmother to granddaughter. The endorsement and avoidance of certain plants and herbs, as well as the use of traditional healers reveals beliefs and behaviours around miscarriage are embedded in cultural matrixes of local healing frameworks and knowledge systems about illness. Findings are consistent with Gerber et al. [56] exploration of the use of complementary and alternative medicine (CAM) among midlife Arab women living in Qatar. Clinical teams should be aware of these healing frameworks to ensure the biomedical health care system complements the health seeking behavior of the Qatari women and recognises their use of traditional healers and medical systems.

Participants’ were engaged with biomedicine and biomedical explanatory frameworks. Most early miscarriages are caused by genetic abnormalities and beyond the control of the pregnant woman, as outlined in the biomedical literature [57–60] and cited by participants. The issue of genetic abnormalities is particularly complex in Qatar due to the prevalence of consanguinity, but only one participant cited cousin marriage as a possible risk and spoke of it specifically in relation to her sister. This woman worked in a school for disabled children and was thus more familiar with discussions of risk and consanguinity. Participants offered a range of biomedical factors as potential causes such as diabetes, “a clot in the placenta”, immunological factors, and uterine abnormalities. The relationship between these factors and the incidence of miscarriage has been documented [61–63].

Women’s understandings of risk associated with advanced maternal age were based mainly on anecdotal evidence. Availability of a positive example was reported as a reassuring factor for women’s perception of advanced maternal age. However, familiarity with the risk associated with advanced maternal age did not alter their decision making in regards to future conception with participants communicating feelings of societal, marital and extended family pressure to have children as well as their own desire to have many children. Such pressure is linked to the societal and religious expectations on women as reproducers in Qatar. It is necessary to understand miscarriage within the context of the emphasis on procreation in pronatalist Qatari society. Qatari women often feel pressure from husbands, extended family members, as well as the state to produce children. Thus, the loss of a pregnancy should be understood against this backdrop to understand the social importance of the pregnancy for the woman and her extended family. The end of a woman’s reproductive experience brings with it potential vulnerability such as a husband acquiring an additional wife, further indicating the centrality of fertility and procreation to Qatari society. This can impact a women’s perception of risk, miscarriage causation, and preventive measures and clinical teams should be aware of the internal and external pressures on women to continue to have children despite potential risks and understand explanations of risk and cause as embedded in these wider cultural frameworks.

Results suggest that socio-cultural environment plays a role in formulating explanations of miscarriage etiology and such explanations are embedded in wider cultural beliefs, which also impact preventive measures taken and health seeking behaviors. Healthcare providers in Qatar are of various ethnic backgrounds [27] as are their patients. The results emphasized the need to be culturally aware when addressing health policy and healthcare needs of culturally diverse populations undergoing reproductive losses. Understanding the local moral world and its impact on understandings of pregnancy and loss is essential to inform policy, improve health care practices and provide more competent care. In particular, it is recommended that the health care providers be aware of the various cultural, religious, and social meaning networks surrounding pregnancy loss, and be open to explore these with their patients. For example, women reported that the framework of God’s will provides meaning and comfort for experience of loss. Being aware of the prevalence of belief in evil eye and its associated risk will allow for better understanding with patients and open dialogue that often is silenced in the clinical environment. Providers should also be aware of women’s avoidance of or use of foods, herbs and traditional medicine as preventative and prescriptive. Familiarity with beliefs about the causes and the preventive measures of miscarriage contributes to the quality of care provided to women during their pregnancy. Health providers can develop perinatal education that is culturally sensitive, with relevant community involvement. Furthermore, awareness of Qatari models of miscarriage explanations will also alert clinical teams that women from other cultural backgrounds will similarly have culturally influenced models of cause.

This paper contributes to the literature through its focus on women’s health and, specifically pregnancy loss in the Middle East. A strength of the research was the use of in-depth qualitative interviews to explore the experiences of 60 Qatari women, whose voices are often not heard in research. Another strength was the use of multiple methods including interviews, observation, participation, which enabled us to capture the complexity of experience. Another strength was that the women were drawn from the Women’s Hospital, which has a diverse patient population. One limitation was that participants were recruited through the Women’s Hospital and most of them were hospitalized for miscarriage management procedure, thus women who had complete miscarriages without medical intervention were not recruited. Another limitation to the study was the partial absence of men’s voices. The intention was to include husbands as primary participants; however, due to the nature of Qatari society and notions of privacy, they were difficult to recruit, either because they or their wives did not want them to participate.

## Conclusion

Medical institutions and training establishments are increasingly acknowledging the presence and importance of medical beliefs and practices outside the biomedical paradigm. Understanding how patients and those around them understand illness and causation is key for health professionals. Healthcare providers in Qatar are of various ethnic backgrounds [27] as are their patients. The results suggest the need to be culturally aware when addressing the healthcare needs of culturally diverse populations undergoing reproductive losses. The ethnographic approach of this research revealed causal explanations of miscarriage are culturally and socially distinct and are embedded in the local moral world in Qatar. Participants suggest a number of possible causes of miscarriage and held complex and layered notions of causality, often with a number of causes sitting alongside one another simultaneously. Miscarriage practices and beliefs are embedded in social, cultural, religious and medical frameworks. Understanding the socio-cultural environment and its impact on notions of causation are essential to inform policy, improve health care practices and provide more competent care. For example, the framework of God’s will provides meaning and comfort for Qatari women’s experience of loss. Aware of the prevalence of belief in Evil eye and its associated risk allows for better understanding of notions of cause, blame and risk. Providers should also be aware of women’s avoidance of or use of foods, herbs and traditional medicine as preventative and prescriptive. Familiarity with understandings about the causes and the preventive measures of miscarriage contributes to the quality of care provided to women during their pregnancy.

## Abbreviations

ART: Assisted reproductive technologies
CAM: Complementary and alternative medicine
IVF: In vitro fertilisation

## Footnotes

1 Consanguineous marriage is the intermarriage of two individuals who have at least one ancestor in common, the ancestor being no more distant than a great-great grandparent.

2 We excluded women suffering from recurrent pregnancy loss (RPL) because RPL is classified as a distinct medical issue/ condition and it is far less common that sporadic miscarriage. Only 1% of women will experience recurrent miscarriage, whereas sporadic miscarriage is very common [64]. The medical treatment of women suffering RPL differs and the emotional, physical and psychological toll is likely to be different. Also, for reasons of ethics we decided to exclude these women due to potential harm.

3 All names are pseudonyms and obvious identifiers have been changed to maintain anonymity.
